# CRISPR-based screening of host factors in veterinary viral infections: from target discovery to host-directed antiviral strategies

**DOI:** 10.3389/fvets.2026.1854467

**Published:** 2026-07-27

**Authors:** Yanfei Liu, Ling Xu, Yongxiang Yin

**Affiliations:** 1 Department of Pathology, Wuxi Maternity and Child Health Care Hospital, Affiliated Women's Hospital of Jiangnan University, Wuxi, Jiangsu, China; 2 Department of Pharmaceutics, School of Pharmacy, Qingdao University, Qingdao, Shandong, China

**Keywords:** CRISPR/Cas9 screening, host factors, host-pathogen interaction, animal viruses, antiviral strategies

## Abstract

Viral infectious diseases in livestock and poultry cause substantial economic losses worldwide and pose persistent zoonotic threats to public health. Elucidating virus-host interactions is essential for understanding viral pathogenesis and developing effective control strategies. In recent years, genome-wide CRISPR/Cas9 functional screening has emerged as a powerful and unbiased high-throughput approach for identifying host dependency and restriction factors. Enabled by the development of species-specific sgRNA libraries, this technology has significantly advanced research in veterinary virology. In this review, we systematically summarize recent progress in CRISPR/Cas9-based screening studies of major livestock and poultry viruses, including foot-and-mouth disease virus (FMDV), swine enteric coronaviruses, African swine fever virus (ASFV), porcine reproductive and respiratory syndrome virus (PRRSV), avian leukosis virus (ALV), and other zoonotic pathogens. We highlight key host factors involved in viral entry, replication, and egress, and integrate these findings to delineate conserved cross-viral dependency networks, such as sialic acid biosynthesis, endosomal–lysosomal trafficking, double-membrane vesicle (DMV) formation, and interferon signaling pathways. Furthermore, we discuss the translational potential of these genomic discoveries for practical agricultural applications, particularly in the development of host-targeted broad-spectrum antivirals and the generation of disease-resistant livestock (e.g., receptor-edited pigs and chickens) through precise genome editing. Finally, we outline future perspectives, including the integration of single-cell transcriptomics and *in vivo* validation, thereby providing a comprehensive framework for advancing disease control and sustainable breeding in animal agriculture.

## 1 Introduction

Viruses are obligate intracellular parasites that rely extensively on host cellular machinery throughout their replication cycle, including receptor recognition, internalization, genome replication, viral protein translation, and progeny virion assembly and release. Host factors, including proteins and non-coding RNAs, can either facilitate viral infection as dependency factors or restrict viral replication as restriction factors. Therefore, systematically identifying these host factors is essential for understanding viral pathogenesis and provides a foundation for development of broad-spectrum antiviral strategies targeting host pathways.

Traditional approaches for identifying host factors, such as yeast two-hybrid assays, immunoprecipitation, and RNA interference (RNAi) screening, are limited by low throughput, high false-positive rates, and significant off-target effects (1, 2). In recent years, CRISPR/Cas9-based functional genomic screening has emerged as a robust approach for unbiased target discovery in virology. Genome-wide CRISPR/Cas9 knockout (KO) screening enables simultaneous functional disruption of thousands of genes through single-guide RNA (sgRNA) libraries covering the entire genome. Combined with high-throughput sequencing and bioinformatics analysis, this approach allows unbiased and systematic identification of host genes involved in viral infection. Compared with RNAi-based screening, CRISPR screening offers higher specificity, more complete gene knockout efficiency, and lower off-target rates, making it a powerful and widely adopted tool for studying virus-host interactions.

Animal infectious diseases continue to pose a major challenge to the sustainable development of global livestock production. Major viral diseases, including foot-and-mouth disease (FMD), African swine fever (ASF), porcine reproductive and respiratory syndrome (PRRS), and avian influenza (AI), cause billions of dollars in economic losses to the livestock industry each year. In addition, several animal viruses, such as influenza virus, Japanese encephalitis virus (JEV), and Rift Valley fever virus (RVFV), have zoonotic potential and pose serious threats to global public health. Despite rapid progress in host factor research for human viruses such as influenza virus, SARS-CoV-2, and HIV, systematic studies on host factors of livestock and poultry viruses have lagged behind. This gap is mainly attributed to the lack of species-specific genome-wide sgRNA libraries for livestock and poultry, incomplete functional annotation of animal genomes, and limited availability of suitable cell lines for large-scale screening. In recent years, however, the development of species-specific CRISPR tools, including the porcine genome-wide CRISPR library (PigGeCKO) (3) and chicken genome-scale sgRNA libraries (4), has accelerated the application of CRISPR screening in livestock and poultry virology.

Although an increasing number of CRISPR-based screening studies have identified host factors involved in veterinary viral infections, the resulting findings remain scattered across different viral systems and experimental platforms. A comprehensive synthesis of these studies is therefore needed to identify shared and virus-specific host dependency pathways and compare screening outcomes across viruses. In this review, we summarize recent advances in CRISPR-based screening for host factors involved in livestock and poultry viral infections, compare major host pathways identified across different viral systems, discuss the limitations and potential biases of current screening approaches, and highlight their implications for antiviral development and disease-resistant breeding.

## 2 CRISPR/Cas9 screening technology: principles and applications

### 2.1 Classification of screening strategies

Genome-wide CRISPR/Cas9 screening can be broadly classified into three major strategies: knockout (CRISPR-KO), activation (CRISPRa), and interference (CRISPRi), each offering distinct advantages for studying virus-host interactions. CRISPR-KO, the most widely used approach, introduces gene loss-of-function mutations through Cas9-mediated double-strand breaks and is effective for identifying host dependency and restriction factors. In contrast, CRISPRa and CRISPRi utilize catalytically inactive Cas9 (dCas9) fused to transcriptional activation or repression domains to modulate gene expression without permanent genome editing, enabling the identification of antiviral restriction factors and the investigation of essential or dose-sensitive genes. The complementary application of these three strategies provides a comprehensive framework for systematically dissecting host factor networks involved in viral infection.

### 2.2 Screening workflow

CRISPR-KO, CRISPRa, and CRISPRi screening share a similar experimental framework, differing mainly in library design and data interpretation. In a typical CRISPR-KO screen, a genome-wide sgRNA library is delivered into target cells at low multiplicity of infection to establish a genotype–phenotype linkage, followed by viral infection and selective pressure to enrich resistant or susceptible cell populations. sgRNA abundance is then quantified by high-throughput sequencing and analyzed using bioinformatics tools to identify candidate host factors, which are subsequently validated through individual gene perturbation experiments. CRISPRa and CRISPRi screening follow a comparable workflow but require cells stably expressing dCas9-based transcriptional activation or repression systems, and their readouts reflect gene overexpression or partial suppression rather than complete knockout. Currently, CRISPR-KO remains the dominant strategy in livestock virus research, while CRISPRa and CRISPRi provide complementary approaches for identifying antiviral restriction factors and essential host genes.

### 2.3 The importance of species-specific libraries

The construction of species-specific sgRNA libraries is a critical prerequisite for applying CRISPR screening to livestock and poultry virus research. Compared with the well-annotated human genome, livestock and poultry genomes differ substantially in gene composition, sequence structure, and regulatory elements. Direct application of human sgRNA libraries to animal systems may lead to incomplete genome coverage and omission of species-specific host factors, thereby reducing the accuracy and reliability of screening results.

In recent years, several species-specific CRISPR libraries have been developed to address this limitation. The porcine genome-scale CRISPR library PigGeCKO contains 85,674 sgRNAs targeting 17,743 protein-coding genes, 11,053 long non-coding RNAs (ncRNAs), and 551 microRNAs, making it one of the most comprehensive porcine CRISPR libraries currently available (3). Another porcine genome-wide library includes 93,859 sgRNAs covering 16,886 protein-coding genes, 25 long ncRNAs, and 463 microRNAs, further expanding available screening tools (5). In addition, the porcine membrane protein-specific library PigMpCKO, which contains 11,052 sgRNAs targeting 1,607 membrane protein-coding genes, is particularly valuable for identifying viral receptors and entry-related host factors. For avian systems, a chicken genome-wide CRISPR library comprising 76,350 sgRNAs has been established, filling a major technical gap in avian virus host factor research (4).

The development of these species-specific CRISPR libraries has significantly accelerated the identification of host factors in livestock and poultry viruses and provides the technical foundation for many of the key discoveries summarized in this review.

## 3 Progress in CRISPR screening research for various viruses

### 3.1 Foot-and-mouth disease virus

Foot-and-mouth disease virus (FMDV), a positive-sense single-stranded RNA virus in the family Picornaviridae, infects cloven-hoofed animals such as cattle, pigs, and sheep and is classified as a highly contagious transboundary animal disease by the World Organisation for Animal Health (WOAH). CRISPR-based screening has recently advanced the understanding of FMDV host dependency factors. Peng et al. (5) performed the first porcine genome-wide CRISPR/Cas9 knockout screening using a library containing 93,859 sgRNAs and identified TOB1 as a critical host factor for FMDV infection. This study revealed that TOB1 regulates viral replication by coordinating interferon (IFN) signaling and epidermal growth factor receptor (EGFR) pathways, where TOB1 deficiency enhances the IFN response and suppresses EGFR downstream signaling, thereby inhibiting FMDV replication.

In addition to genome-wide screening, several single-gene CRISPR knockout studies have provided complementary evidence for FMDV host regulation. HDAC9 knockout enhances viral replication, indicating a restrictive role mediated through epigenetic regulation (6), while disruption of the WD40 domain of ATG16L1 promotes FMDV infection by affecting non-canonical autophagy (7). Similarly, IRF3 knockout weakens the type I IFN response and increases viral replication in BHK-21 cells, further confirming the central role of the interferon pathway in FMDV infection (8). Collectively, these studies highlight the importance of interferon signaling, EGFR-mediated pathways, and autophagy-related processes in regulating FMDV-host interactions. In contrast to several veterinary viruses, including PEDV, PDCoV, JEV, RVFV, and ASFV, whose CRISPR hits are enriched in viral entry and trafficking pathways, FMDV studies have primarily highlighted intracellular immune regulation and autophagy-related mechanisms.

### 3.2 Swine enteric coronavirus

Swine enteric coronavirus (SECoVs), including porcine epidemic diarrhea virus (PEDV), porcine deltacoronavirus (PDCoV), transmissible gastroenteritis virus (TGEV), and swine acute diarrhea syndrome coronavirus (SADS-CoV), have caused high mortality in piglets and, therefore, pose serious threats to the pork industry (9).

#### 3.2.1 Porcine epidemic diarrhea virus

Porcine epidemic diarrhea virus (PEDV), a member of the genus Alphacoronavirus in the family Coronaviridae, is a major enteric pathogen in swine, causing severe diarrhea and up to 100% mortality in neonatal piglets. In recent years, multiple genome-wide CRISPR screening studies have systematically uncovered the host factor network required for PEDV infection. Genome-wide CRISPR screening in Huh7 cells revealed that the sialic acid biosynthesis pathway and the heparan sulfate synthesis pathway are essential host dependency factors for PEDV infection, highlighting the critical role of cell surface glycans in viral attachment and entry (10). Subsequent screening using the VeroCKO library further confirmed SLC35A1 as a key factor, demonstrating that disruption of sialic acid transport significantly impairs PEDV infection (11). These findings collectively establish glycan-mediated attachment as a core mechanism shared among several porcine coronaviruses. Additional CRISPR screening studies have identified multiple host regulators involved in PEDV entry and replication. IFITM3 was found to facilitate PEDV entry, suggesting that PEDV may hijack IFITM-mediated membrane fusion mechanisms despite the traditional antiviral role of IFITM proteins (12). Genome-wide screening in porcine intestinal epithelial cells identified YIPF5 as an essential factor for replication by regulating the formation of double-membrane vesicles (DMVs), which are critical for coronavirus RNA synthesis (13). Using a membrane protein-specific CRISPR library (PigMpCKO), RPSA was identified as a key host factor, indicating that PEDV may utilize conserved cell surface receptors shared by different viruses (14). In addition, PKCθ was shown to regulate PEDV replication, providing further insight into signal transduction mechanisms during infection (15). Overall, CRISPR screening studies have revealed that PEDV infection relies on a coordinated host network involving glycan biosynthesis, membrane fusion factors, replication organelle formation, and signaling pathways. Notably, the recurrent identification of SLC35A1 in both PEDV and PDCoV screens indicates that sialic acid metabolism is a conserved host dependency pathway among swine coronaviruses, paralleling the central role of glycan pathways in influenza virus infection.

#### 3.2.2 Porcine deltacoronavirus

Porcine deltacoronavirus (PDCoV) is an emerging enteric coronavirus with potential cross-species transmission risk. Compared with PEDV, belongs to a different coronavirus genus, yet CRISPR-based screens indicate that these viruses converge on partially overlapping host pathways, particularly glycan-mediated attachment process. Genome-wide CRISPR screening in LLC-PK1 cells identified SLC35A1, a CMP-sialic acid transporter, as a critical host factor for PDCoV adsorption, supporting the conserved role of the sialic acid biosynthesis and transport pathway in porcine coronavirus entry (16). This finding is consistent with PEDV screens in which disruption of sialic acid-related pathways markedly impaired infection. Beyond viral adsorption, additional screens have revealed PDCoV-specific dependencies involved in intracellular replication. C16orf62 was identified as a host dependency factor associated with attachment and internalization (17), while HSP90AB1 was shown to promote viral protein from degradation (18). Notably, HSP90AB1 has also been reported as a host factor that enhances TGEV infection, suggesting that this chaperone may represent a conserved post-entry dependency across multiple porcine enteric coronaviruses (19). These findings indicate that PDCoV relies on both entry-associated and post-entry host pathways.

#### 3.2.3 Transmissible gastroenteritis virus and swine acute diarrhea syndrome coronavirus

Beyond PDCoV, CRISPR screening has also uncovered conserved mechanisms across coronaviruses. TMEM198 was identified as a key regulator of double-membrane vesicle (DMV) formation in multiple coronaviruses, suggesting a common mechanism for replication organelle biogenesis (20). Similarly, PLAC8 was found to be essential for SADS-CoV infection, providing additional evidence that coronaviruses rely on specific host factors to mediate cell entry and replication (21). Overall, CRISPR-based studies indicate that PEDV, PDCoV, TGEV and SADA-CoV infection depend on coordinated regulation of glycan-mediated attachment, endoplasmic reticulum protein folding, replication complex assembly, and DMV formation, highlighting conserved host pathways that may serve as broad-spectrum antiviral targets.

### 3.3 African swine fever virus

African swine fever virus (ASFV) is a large nucleocytoplasmic double-stranded DNA virus whose 170–190 kb genome encodes more than 150 viral proteins. ASFV causes a highly lethal disease in domestic pigs, with mortality rates that can approach 100%. Recent CRISPR screening studies performed in wild boar lung cells have begun to identify host factors and pathways involved in ASFV infection, although the physiological relevance of these findings to infection in primary porcine macrophages remains to be established. Genome-wide CRISPR screening using a porcine sgRNA library identified TMEM239 as an essential host factor for ASFV entry, where TMEM239 promotes viral internalization by regulating early endosome function (22). Another CRISPR screening study revealed that MHC class II-related molecules, including RFXANK, RFXAP, SLA-DMA, SLA-DMB, and CIITA, are required for efficient ASFV replication, suggesting that ASFV may exploit antigen presentation-associated endocytic pathways during infection (23). These findings highlight the importance of endosomal trafficking and immune-related pathways in virus-host interactions. Compared with RNA viruses such as FMDV and PRRSV, ASFV screens have highlighted a distinct enrichment of endosomal and immune associated host factors, possibly reflecting the complex entry route and replication strategy of this large DNA virus.

In addition to host factor identification, CRISPR-related technologies have also facilitated ASFV research tools. A recombinant ASFV carrying dual reporter genes (luciferase and eGFP) has been developed for high-throughput antiviral screening, providing a valuable platform for drug discovery and functional studies (24). Collectively, CRISPR-based approaches are progressively revealing the complex host regulatory network required for ASFV entry, replication, and immune evasion, offering new opportunities for antiviral target identification and therapeutic development.

### 3.4 Porcine reproductive and respiratory syndrome virus

Porcine reproductive and respiratory syndrome virus (PRRSV) is one of the most economically important viral pathogens in the global swine industry, causing substantial reproductive failure and respiratory disease in pigs. PRRSV exhibits strict cell tropism, primarily infecting monocyte/macrophage lineage cells, and the scavenger receptor CD163 has been well established as the essential entry and fusion receptor for viral infection (25).

Genome-wide CRISPR/Cas9 screening has further expanded the understanding of PRRSV-host interactions. Using a whole-genome CRISPR library in PK15 cells, Jiang et al. (26) systematically identified host factors involved in PRRSV replication, including KXD1, PSMD3, and LGALS2. Subsequent mechanistic studies demonstrated that KXD1 depletion restricts PRRSV replication by regulating autophagy. By contrast, lysosomal-associated transmembrane protein 4A (LAPTM4A) has been identified as a critical restriction factor that maintains lysosomal homeostasis and promotes complete autophagic flux, thereby limiting PRRSV infection (27). Together, these findings underscore the multifaceted roles of autophagy-related pathways in PRRSV infection.

Building on the critical role of CD163, CRISPR/Cas9-mediated gene editing has enabled the generation of PRRSV-resistant pigs through targeted knockout or precise modification of the SRCR5 domain of the CD163 gene (28). These gene-edited pigs exhibit complete resistance to PRRSV infection without detectable adverse effects on growth or immune function, representing a major breakthrough in disease-resistant livestock breeding. This success demonstrates the translational potential of CRISPR-based host factor research, bridging mechanistic studies and practical applications in antiviral breeding and disease control.

### 3.5 Influenza virus

Influenza virus is one of the most thoroughly studied viruses and the most widely used viral model for CRISPR whole-genome screening, accumulating a large amount of high-quality host factor data.

Multiple independent CRISPR screening studies have consistently identified the sialic acid biosynthesis and transport pathway (including GNE, NANS, CMAS, SLC35A1, and SLC35A2) as the most critical host dependency network for influenza virus infection. Influenza virus initiates infection through hemagglutinin-mediated binding to sialic acid receptors on the cell surface, and disruption of this pathway fundamentally blocks viral entry. Further studies demonstrated that host regulators such as STK11 and members of the COG complex (COG6 and COG8) modulate influenza infection by controlling sialic acid receptor distribution and glycosylation in the Golgi apparatus, highlighting the importance of glycan biosynthesis and vesicle transport in viral attachment (29–31).

In addition to receptor-related pathways, CRISPR screening has identified multiple host factors involved in endocytosis, membrane trafficking, and viral replication. CYTH2 and IGDCC4 were shown to regulate influenza virus internalization and endosomal escape, revealing key mechanisms underlying viral entry and intracellular trafficking (32, 33). Immune-related host factors have also been uncovered, including IFIT2 (34), which can be hijacked to promote viral mRNA translation, and JADE3 (35), which enhances antiviral resistance by activating NF-κB-dependent IFITM3 expression, demonstrating the dual role of host immune regulators in influenza infection.

CRISPR screening has further provided insights into cross-species transmission and host adaptation. Using a genome-wide CRISPR screen in porcine kidney cells, Zou et al. identified GGCX as a host dependency factor for Eurasian avian-like H1N1 swine influenza virus (EA H1N1 SIV) and showed that GGCX promotes receptor-binding adaptation associated with interspecies transmission (36). Meta-analysis of published influenza CRISPR screening studies indicates limited overlap among datasets, emphasizing that cell type, viral strain, and experimental conditions significantly influence host factor identification and highlighting the importance of multi-model screening strategies (37). Recent studies have shown that CRISPR activation screening identified cholesterol 25-hydroxylase (CH25H) as an influenza virus-inducible restriction factor that inhibits viral membrane fusion (38). Notably, the repeated identification of glycan biosynthesis factors in influenza screens mirrors observations in PEDV, PDCoV, and RVFV, suggesting that host glycan availability is a recurrent determinant of viral attachment across multiple veterinary viruses. Overall, CRISPR-based studies demonstrate that influenza virus infection relies on coordinated regulation of glycan biosynthesis, vesicle transport, endosomal trafficking, immune signaling, and host adaptation pathways, making influenza virus a representative model for understanding universal principles of virus-host interactions.

### 3.6 Avian leukosis virus

Avian leukosis virus (ALV) is an oncogenic retrovirus that poses a major threat to the global poultry industry, with subgroup J (ALV-J) being the most pathogenic and economically damaging strain. So far, only one study has used a chicken whole-genome CRISPR/Cas9 library in DF-1 cells to identify ALV-J host dependency factors, revealing dozens of host factors involved in viral replication. Among these, Cables1 was shown to promote ALV-J replication by regulating cell cycle progression and intracellular signaling pathways (39). In addition to host factor identification, CRISPR/Cas9-mediated gene editing has enabled the development of ALV-J-resistant chickens by targeting the viral receptor NHE1 (40). Precise deletion of the tryptophan residue at position 38 (W38) in NHE1 completely blocks ALV-J entry without affecting normal physiological function, demonstrating the feasibility of receptor-based antiviral breeding strategies. However, subsequent studies showed that ALV-J can overcome this receptor barrier through rapid adaptive evolution, indicating that single-target gene editing strategies may be vulnerable to viral escape and highlighting the necessity of multi-target or combinatorial resistance approaches. Overall, CRISPR-based studies on ALV illustrate both the mechanistic value of host factor screening and the practical potential of gene editing in disease-resistant poultry breeding.

### 3.7 Japanese encephalitis virus

Japanese encephalitis virus (JEV) is a mosquito-borne zoonotic flavivirus that causes reproductive disorders in pigs and severe viral encephalitis in humans, posing a significant threat to both animal health and public health in Asia. Using the porcine whole-genome CRISPR library PigGeCKO, researchers identified host factors that facilitate JEV infection in porcine cells, including genes involved in heparan sulfate proteoglycan sulfation, endoplasmic reticulum membrane protein complex 3 (EMC3), endoplasmic reticulum membrane protein complex 6 (EMC6) and calcium homeostasis (CALR) (3). These factors are consistent with a model in which JEV binds to HSPGs and/or other cellular receptors to enter cells, and then relies on ER-associated components to support replication.

Subsequent mechanistic studies further clarified key host regulators identified through CRISPR screening. RAB4B, a small GTPase involved in early endosomal trafficking, was shown to directly interact with the JEV envelope protein and promote viral entry, while its knockout significantly inhibited infection (41, 42). In addition, ZNF33B was found to enhance JEV RNA stability and replication by regulating HSPB1/8-mediated SUMOylation of the viral NS5 protein, revealing a novel post-translational regulatory mechanism (43). These findings place JEV alongside other veterinary viruses, such as ASFV, RVFV, and swine coronaviruses, in highlighting the importance of vesicular and endosomal trafficking during infection.

### 3.8 Rift Valley fever virus

Rift Valley fever virus (RVFV) is a mosquito-borne zoonotic pathogen belonging to the genus Phlebovirus in the family Phenuiviridae (order Bunyavirales). It causes large-scale abortions in ruminants and hemorrhagic fever in humans and is listed by the World Health Organization as a priority pathogen with epidemic potential. CRISPR-based genome-wide screening has provided important insights into the host factors required for RVFV infection.

Genome-wide CRISPR screening identified LRP1, Grp94 and RAP, with mechanistic studies demonstrating that the RVFV glycoprotein Gn directly binds to a specific domain of LRP1, establishing LRP1 as a functional entry receptor independent of glycosylation modification (44). Another study also confirmed LRP1 as a dependency factor for RVFV and additionally identified WDR7 as a host factor required for infection, although the underlying mechanism remains unclear (45).

In addition, an earlier haploid genetic screening also identified glycosaminoglycan (GAG) biosynthesis genes, including members of the COG complex, suggesting that heparan sulfate proteoglycans contribute to RVFV adsorption (46). These studies indicate that RVFV infection relies on coordinated regulation of receptor-mediated entry and glycan-dependent adsorption, providing a systematic understanding of RVFV-host interactions and potential antiviral targets.

### 3.9 Bovine parainfluenza virus type 3

Bovine parainfluenza virus 3 (BPIV3) is an important respiratory pathogen in cattle and a component of the bovine respiratory disease complex. A recent genome-scale CRISPR screen in BPIV3 identified multiple host factors associated with viral infection, including SLC35A1 and the newly discovered WNT5A, SELENON, and SLC16A13, which were shown to be critical regulators of BPIV3a replication, as well as LSM12, which was further implicated in the viral entry process (47, 48). These studies uncover broader virus-host interaction networks in cattle.

## 4 Cross-viral common host factor networks

### 4.1 Sialic acid biosynthesis and transport pathway

The sialic acid pathway represents the most consistently enriched host dependency network identified across CRISPR screens of multiple livestock viruses, including influenza virus, PEDV, and PDCoV. Sialic acid residues, located at the termini of cell surface glycans, serve as critical attachment receptors for diverse viruses. Disruption of key components in this pathway, such as SLC35A1, effectively abolishes viral entry, underscoring its central role in infection.

Despite sharing a common dependency, viruses exploit sialic acid through distinct mechanisms. Influenza virus directly binds α2,3- or α2,6-linked sialic acid via hemagglutinin, whereas coronaviruses such as PEDV and PDCoV utilize spike proteins to facilitate initial attachment. Beyond biosynthesis, host regulators including STK11 modulate the spatial organization of sialic acid on the cell surface, revealing additional regulatory layers (30). The evolutionary conservation of this pathway highlights its potential as a target for broad-spectrum antiviral strategies, although therapeutic targeting must balance antiviral efficacy with host toxicity.

### 4.2 Endosomal-lysosomal pathway

The endosomal-lysosomal system is a central hub for viral entry, uncoating, and genome release and has been repeatedly identified in CRISPR screens of ASFV, influenza virus, JEV, and RVFV. Key components include RAB GTPases (e.g., RAB4B, RAB5, RAB7), which regulate vesicular trafficking and maturation, as well as V-ATPase, which maintains the acidic environment required for membrane fusion.

Viruses exhibit both shared and distinct dependencies on this pathway. ASFV utilizes early endosomes regulated by TMEM239, JEV exploits RAB4B-mediated endosomal recycling through direct interaction with its envelope protein, and RVFV enters via LRP1-dependent endocytosis followed by acidification-driven fusion (22, 41, 45). Influenza virus relies on host regulators such as CYTH2 to facilitate endosomal escape (33). The requirement for endosomal acidification represents a common vulnerability, explaining the broad antiviral activity of lysosomotropic drugs, although their clinical application is limited by cytotoxicity.

### 4.3 Double-membrane vesicle formation

Double-membrane vesicles (DMVs) are specialized replication organelles formed by coronaviruses through extensive remodeling of the endoplasmic reticulum. CRISPR screening has identified conserved host factors involved in DMV biogenesis, including YIPF5 and TMEM198, highlighting a shared mechanism across coronavirus genera.

YIPF5, an ER-Golgi intermediate compartment protein, is specifically required for PEDV-induced DMV formation without disrupting general secretory pathways, suggesting a virus-specific role in membrane remodeling (13). Similarly, TMEM198 has been identified as an essential factor for DMV formation in both alpha- and beta-coronaviruses, indicating a conserved requirement across divergent coronaviruses (20). These findings suggest that targeting DMV biogenesis may represent a selective and potentially broad-spectrum antiviral strategy against coronaviruses.

### 4.4 Golgi apparatus and glycoprotein processing (COG complex)

The conserved oligomeric Golgi (COG) complex is essential for maintaining Golgi structure and vesicle trafficking fidelity and has emerged as a recurrent host dependency factor in CRISPR screens of influenza virus and RVFV. The complex regulates retrograde vesicular transport and ensures proper localization of glycosyltransferases, thereby maintaining the integrity of cell surface glycosylation, including sialic acid receptor expression.

Disruption of COG components (e.g., COG6, COG8) impairs glycoprotein processing and reduces surface sialylation, thereby inhibiting viral attachment. In addition, the COG complex contributes to the maturation of viral glycoproteins, such as influenza hemagglutinin and RVFV envelope proteins (29, 31, 46). These findings highlight a functional intersection between glycan biosynthesis and intracellular trafficking pathways, emphasizing the integrated nature of host dependency networks exploited by viruses.

### 4.5 Interferon signaling pathway

The interferon (IFN) signaling pathway constitutes the core of host antiviral innate immunity, yet CRISPR screening reveals that its components can play virus-specific and even opposing roles. In FMDV infection, TOB1 acts as a negative regulator of IFN signaling, and its disruption enhances antiviral responses, thereby restricting viral replication (5). In contrast, in influenza virus infection, factors such as JADE3 promote antiviral activity by enhancing NF-κB–dependent IFITM3 expression (35).

Notably, IFITM3 exhibits a dual role: it acts as a restriction factor in influenza virus infection by blocking membrane fusion (49), but paradoxically facilitates entry in PEDV infection (12). This functional divergence highlights the complexity of host-virus interactions and suggests that viruses can repurpose antiviral proteins to support their own life cycle. These findings underscore that while IFN signaling is broadly antiviral, its specific regulatory outcomes are highly virus-dependent, presenting both opportunities and challenges for host-targeted antiviral strategies.

### 4.6 Receptor-mediated cell pathway

Receptor-mediated cell pathway is the most shared category of host factors (involving 7 viruses), but the specific receptor molecules used by each virus are highly specific, reflecting the selective recognition strategies of different viruses on host cell surface molecules (Table 1).

**Table 1:** Key receptor identified by CRISPR screening for important livestock and poultry viruses.

Virus	Key receptors	Function
PEDV	RPSA	Attachment factor
PDCoV	SLC35A1	Sialylation factor
ASFV	TMEM239	Entry cofactor
Influenza virus	SLC35A1	Sialylation factor
ALV-J	NHE1	Functional receptor
RVFV	LRP1	Entry receptor
BPIV3	SLC35A1	Sialylation factor

The specificity of receptor factors makes them ideal targets for gene editing in disease-resistant breeding-precisely editing the viral binding site of the receptor can confer genetic resistance to specific viruses in animals without affecting the normal physiological function of the receptor. However, the ALV-J case serves as a warning that viruses can overcome the barrier of a single receptor through rapid adaptive evolution, and a multi-target combined editing strategy is a necessary direction for improving disease resistance stability.

## 5 Limitations and potential biases of CRISPR screening

While CRISPR screening has revolutionized the identification of host factors involved in viral infection, it is imperative to acknowledge that no methodology is without inherent constraints. Many screens have the limitation that genes conferring mild phenotypes or those essential for growth can be overlooked, as every genetic perturbation is incorporated in the same population.

Conventional CRISPR-KO screens are intrinsically limited in their ability to identify essential host genes. Disruption of genes required for cell survival or proliferation can result in premature depletion of mutant cells from the screening population, preventing their detection as candidate host factors (50, 51). Consequently, essential cellular genes that may play critical roles during viral infection can be systematically underrepresented in screening datasets.

Another important caveat is that gene knockout can trigger compensatory mechanisms that mask the true phenotype of gene loss, leading to both false negatives and, in some cases, false identification of hits. Genetic compensation occurs when the loss of a gene activates the upregulation of compensatory genes, often through transcriptional adaptation triggered by premature termination codon-containing mutant mRNA (52).

Host factor requirements are highly dependent on cellular context. Most veterinary virus CRISPR screens have been conducted in immortalized cell lines such as PK15, DF-1, Vero, Huh7 and LLC-PK1 cells because of their amenability to genome editing and large-scale screening. However, these models may not fully recapitulate the physiological environment of primary target cells *in vivo*. For example, PRRSV primarily infects porcine alveolar macrophages, whereas many genome-wide screens have been performed in PK15 cells. Similarly, ASFV naturally targets monocytes and macrophages, yet available screens frequently utilize WSL cells. Host factor dependencies identified by CRISPR screening can differ markedly across cell types and experimental systems, underscoring the need to validate candidate genes in physiologically relevant primary cells or *in vivo* models.

Technical limitations associated with sgRNA library design should also be considered. Although genomic resources for major livestock and poultry species have improved substantially in recent years, their functional annotation and CRISPR screening toolkits remain less mature than those available for human and mouse systems. Consequently, species-specific CRISPR libraries may vary in genome coverage, sgRNA quality, and representation of alternatively spliced transcripts. Incomplete annotation can lead to missed host factors, whereas uneven sgRNA distribution may reduce screening sensitivity and reproducibility.

Finally, although CRISPR screening generally exhibits greater specificity than RNA interference-based approaches, off-target cleavage and variable sgRNA activity remain important concerns (53, 54). Candidate genes identified through pooled screening should therefore be independently validated using multiple sgRNAs, complementary gene perturbation approaches, and orthogonal experimental systems to ensure biological robustness.

## 6 Summary and outlook

**Figure 1:**
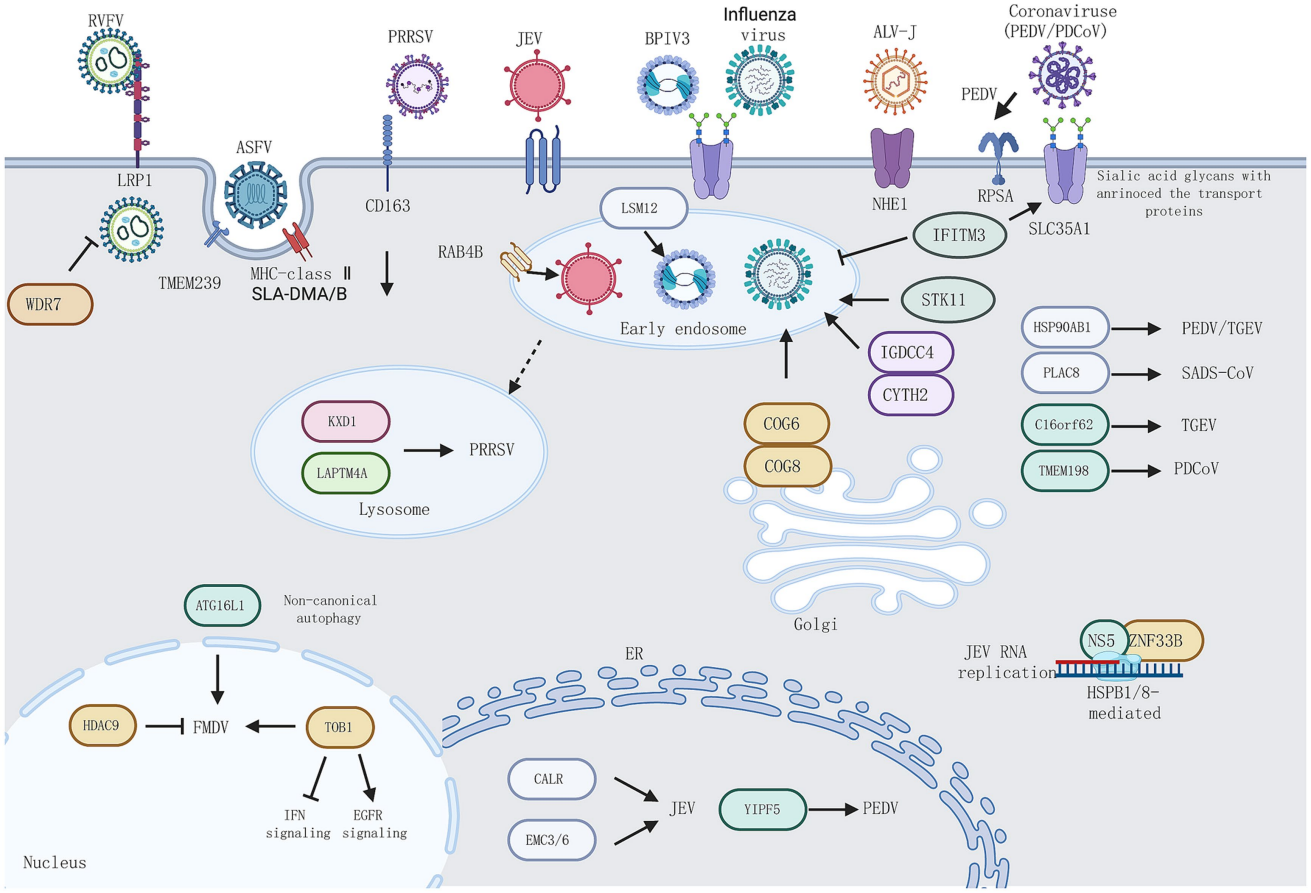
Landscape of host dependency factors in animal viruses identified by CRISPR screening. Schematic overview of cellular proteins and pathways that regulate infection by multiple animal viruses. At the plasma membrane, viral attachment and entry are mediated by host receptors or cofactors, including LRP1 for RVFV, TMEM239 and MHC class II molecules for ASFV, CD163 for PRRSV, NHE1 for ALV-J, and RPSA/SLC35A1-associated sialylated glycans for PEDV and related coronaviruses. Following internalization, viruses traffic through early endosomes and lysosomes, where factors such as RAB4B, LSM12, IFITM3, STK11, IGDCC4, CYTH2, KXD1, and LAPTM4A modulate endosomal maturation, restriction, or viral progression. ER- and Golgi-associated proteins, including CALR, EMC3/6, YIPF5, COG6, and COG8, participate in viral replication, assembly, or intracellular transport. Additional host factors regulate specific viral infections, including HSP90AB1 for PEDV/TGEV, PLAC8 for SADS-CoV, C16orf62 for TGEV, TMEM198 for PDCoV, and the HSPB1/8-mediated NS5–ZNF33B axis during JEV RNA replication. Nuclear and autophagy-related regulators, such as ATG16L1, HDAC9, and TOB1, influence FMDV infection through non-canonical autophagy and antiviral or growth factor signaling pathways. Arrows indicate proviral or required functions, whereas blunt-ended lines indicate inhibitory effects. The image is drawn using Biorender software.

CRISPR-based genome-wide screening has emerged as a powerful platform for systematically identifying host factors that govern viral infection in livestock and poultry. Substantial progress across diverse viruses, including PEDV, ASFV, PRRSV, influenza virus and JEV, has revealed conserved host dependency networks, particularly those involving sialic acid metabolism, endosomal trafficking, and replication organelle formation (Figure 1). These findings not only deepen our understanding of virus-host interactions but also provide a molecular foundation for the development of host-targeted, broad-spectrum antiviral strategies.

Importantly, key receptor and replication-associated factors identified through CRISPR screening, such as NHE1, has enabled the successful generation of disease-resistant livestock, demonstrating a clear translational pipeline from mechanistic discovery to practical application. This integration of functional genomics and gene editing represents a paradigm shift in the control of infectious diseases in animal agriculture.

Looking ahead, several directions will further advance this field. These include improving species-specific sgRNA libraries for major livestock animals, integrating CRISPR screening with single-cell transcriptomics to resolve functional heterogeneity, performing comparative multi-virus screens to distinguish conserved and virus-specific host dependencies, and strengthening *in vivo* validation to bridge the gap between cell-based screening and animal-level phenotypes. In parallel, translating CRISPR discoveries into disease-resistant breeding programs will require careful consideration of biosafety, regulatory frameworks, and ethical issues.

Overall, continued innovation in CRISPR screening technologies and their integration with multi-omics approaches will provide deeper insights into viral pathogenesis and accelerate the development of effective strategies for the prevention and control of major livestock and poultry diseases.
